# A Case Report of Melanoma as Acute Mastoiditis in a 10-Month-Old Female Child

**DOI:** 10.1155/2019/9641945

**Published:** 2019-02-14

**Authors:** Konstantina Chrysouli, Michael Tsakanikos, Sofia Stamataki

**Affiliations:** ENT Department, “P. & A. Kyriakou” Children's Hospital of Athens, Athens, Greece

## Abstract

We present a rare case of transplacental-transmitted maternal melanoma to the placenta and foetus during the pregnancy of a 34-year-old woman. She was diagnosed with a melanoma at the age of 25, for which she was treated with chemotherapy. During her pregnancy, she presented with a recurrence of the disease and died 3 months after delivery. The 10-month-old female child presented with a recurrent retroauricular oedema on the left side. A trephination of mastoid apophysis followed. Multiple fragments of dark-coloured tissue were sent for histological examination, and the immunophenotype showed a melanocytic tumour in the mastoid. A full radiological assessment showed no sign of metastasis. The child remained without treatment. Complete remission of bone metastatic lesion has been confirmed by follow-up; now, the child is 4 years old, alive, and without evidence of disease.

## 1. Introduction

Foetal metastases are rare. Melanoma is the most common neoplasm with transplacental transmission to the foetus with very poor prognosis [1, 2]. We present a rare case with transplacental-transmitted metastases from maternal melanoma to the mastoid with spontaneous regression.

## 2. Case Report

**Figure 1:**
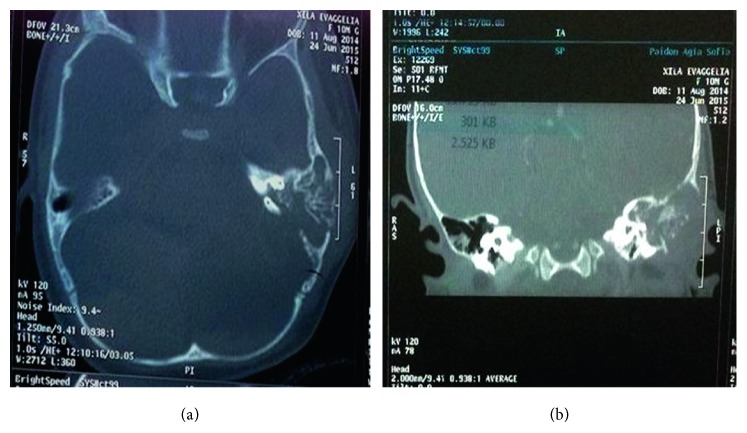
CT of the temporal bone. An invasive lesion of the mastoid cavity on the left with widespread corrosion of the trabeculae of the bone expanding towards the cranial bones and the underlying meninx.

**Figure 2:**
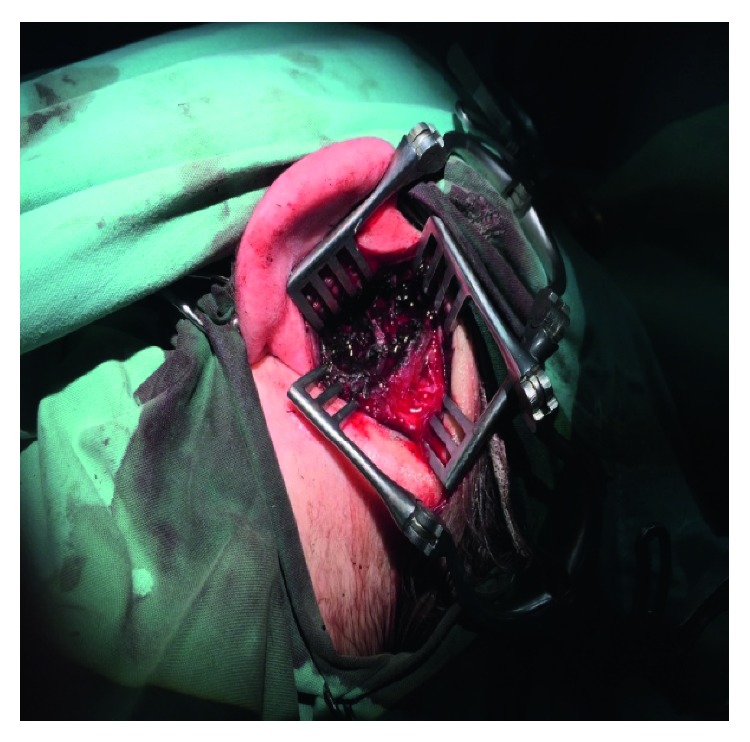
Α retroauricular incision. Ιnfiltration with multiple friable fragments of dark-coloured subcutaneous tissue (a material similar to cuttlefish ink in colour) of the whole mastoid cavity, which had been submitted to “automated trephination.” Drilling of the mastoid with debridement and removal of the corroded bone fragments.

A 10-month-old female presented with an oedema in the left zygomatic and retroauricular region without other inflammation sites. She was afebrile and in good clinical condition with otoscopic findings, characteristic of acute otitis media and concomitant oedema in the external auditory meatus of the left ear. Due to otorrhoea on the left side 4 days ago, the child started receiving antibiotic treatment per os with amoxicillin and clavulanic acid 457 mg/5 ml (90 mg/kg) every 12 h. Family history showed that the mother died 7 months ago at the age of 34 due to melanoma recurrence during pregnancy. She was diagnosed with melanoma at the age of 25, for which she was treated with chemotherapy with complete regression of the disease. During her pregnancy, she presented with a recurrence of melanoma with metastases in the liver, bones, lungs, and brain. She died 3 months after delivery. The child was initially treated as an acute mastoiditis on the left side according to our clinic's protocol, and a double intravenous antibiotic scheme of cefotaxime + clindamycin and dexamethasone was administered. Subsequently, a myringotomy was performed on both sides under general anaesthesia, and ventilation tubes were placed. A purulent fluid was drained from the left side, which was sent for culture. The child showed an immediate improvement in her clinical picture, showing reduced otorrhoea on the left and reduced oedema in the left zygomatic and retroauricular region after the following 24 hours. After the antibiogram results (*Pseudomonas aeruginosa*), the treatment was changed to ceftazidime and amikacin. Due to recurrence of the retroauricular oedema on the left after 7 days, a CT of the temporal bone with contrast was performed. An invasive lesion of the mastoid cavity on the left with widespread corrosion of the trabeculae of the bone was found, expanding intracranially (towards the cranial bones and the underlying meninx) (Figure 1). A drilling of the mastoid on the left followed. During the retroauricular incision, an infiltration was observed, with multiple friable fragments of dark-coloured subcutaneous tissue of the underlying corroded bone cortex and of the whole mastoid cavity, which had been submitted to “automated trephination.” Characteristically, the mastoid cavity was infused with a material similar to cuttlefish ink in colour (Figure 2). Moreover, corrosion was observed on the posterior wall of the external auditory meatus, on the apex of the mastoid, and on the bony wall of the meninx, which was uncovered especially in the area of the meninx-sigmoid corner. Furthermore, the wall of the sigmoid sinus was corroded. No thrombosis was observed of the sigmoid sinus. Neuromonitoring of the facial nerve was performed, and an urgent neurosurgical assessment was requested. Debridement and removal of the corroded bone fragments was performed.

**Figure 3:**
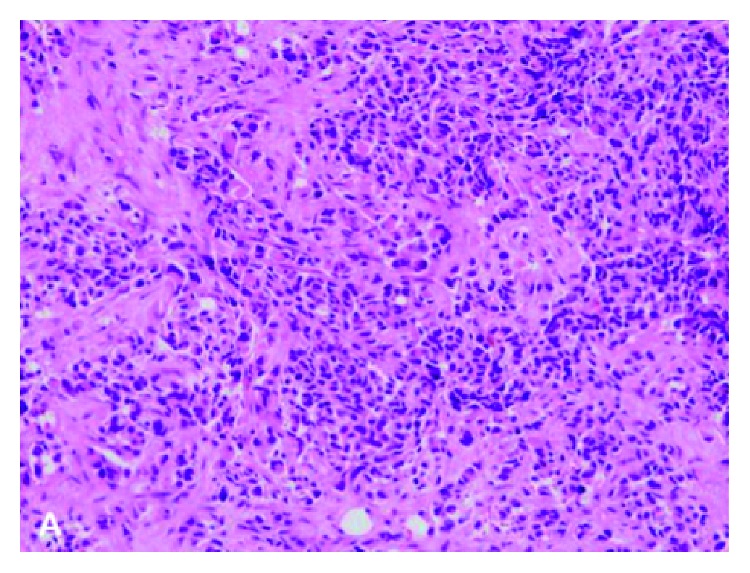
Foetal metastases in the mastoid. The biopsy shows a tumour consisting of nests of small-sized and medium-sized cells, with oval to round nuclei and poorly defined eosinophilic cytoplasms (hematoxylin-eosin, original magnification 200x).

Multiple fragments of dark-coloured tissue were sent for an immediate histological examination. The history (individual and family), the clinical picture, the radiological and surgical findings, and the immunophenotype showed an intermediate level malignity of a melanocytic tumour in the mastoid, with areas of a high level of malignity (Figure 3). Oncologists were consulted, and we came into communication with the international “rare tumours” protocol in order to choose the right therapy. Using the real-time PCR-HRM analysis technique, a mutation was detected in exon 15 of the ΒRAF (p.V600E) gene. A full radiological examination was followed by an MRI of the brain, an MRI of the visceral cranium, and an MRI of the vertebral column; a thorax-CT; a cervical/parotid/axillary/groin U/S; and an upper-lower abdomen U/S.

**Figure 4:**
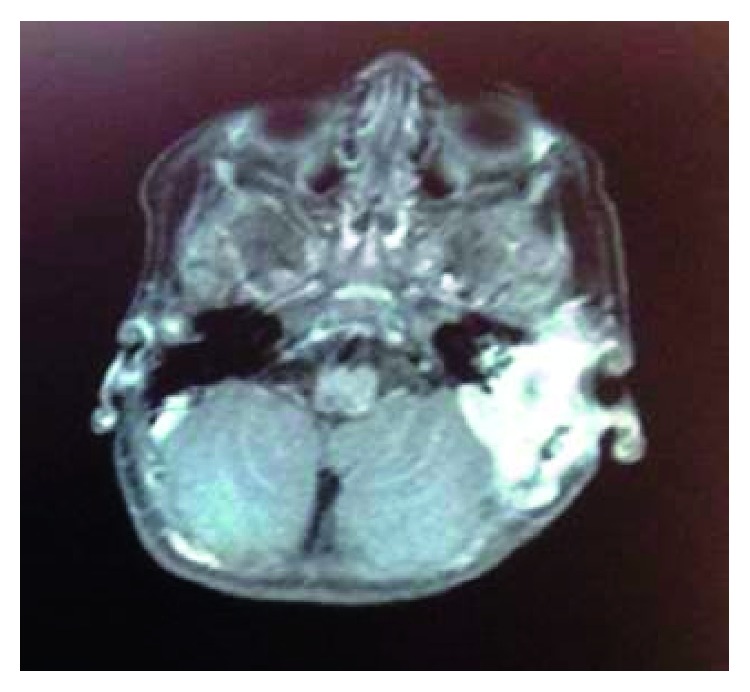
First MRI: pathological magnetic signal with mild enhancement by contrast media and areas of necrosis of all cavities of the temporal bone. Whether this lesion corresponds to a residual disease or postoperative lesions remains a question

The visceral cranium MRI showed an invasive lesion of a pathological magnetic signal with mild enhancement by contrast administration and areas of necrosis, occupying all cavities of the left temporal bone (Figure 4). Whether this lesion corresponds to a residual disease or postoperative lesions remains a question.

The rest of the radiological examination was normal. The child remained in good clinical condition without treatment.

**Figure 5:**
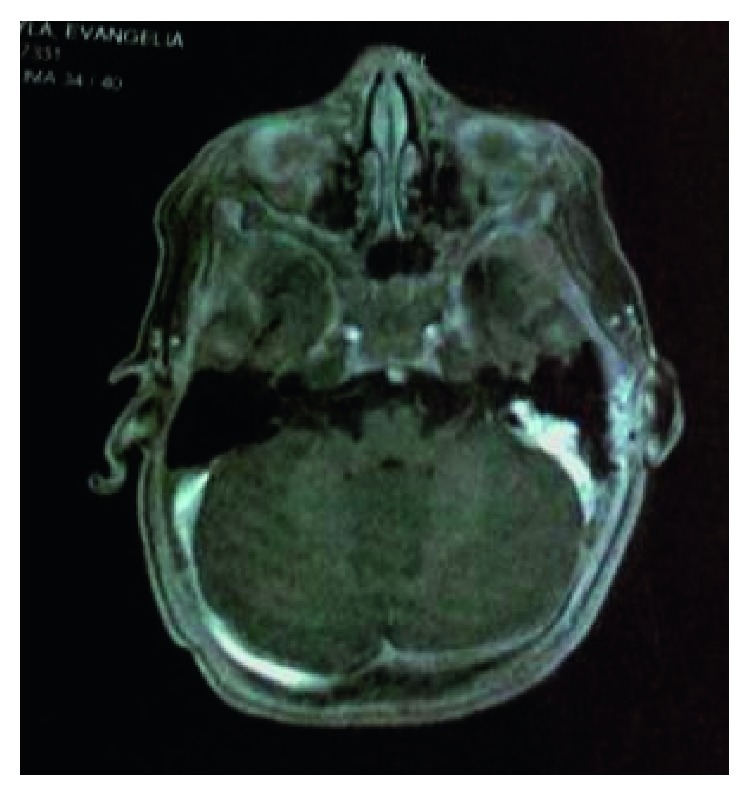
Second MRI (one month later): clear reduction in the area of the pathological signal and of the intake of paramagnetic substance in and around the left temporal bone.

One month later, due to the appearance of asymmetry on the cheek and the right preauricular region, second visceral cranium MRI was performed, which was negative for activation of the disease. Compared to the previous ΜRI, we distinguished a clear reduction in the area of the pathological signal and of the intake of the paramagnetic substance in and around the left temporal bone (Figure 5). Complete regression of the metastatic bone lesion has been confirmed by follow-up; now, the child is 4 years old, alive, and without evidence of disease.

## 3. Discussion

In a comprehensive search of the MEDLINE database (1966 to 2002), Alexander et al. [3] found that only 87 cases of placental or foetal metastases have been reported.

Melanoma most frequently involves the placenta or the foetus. There are 27 of 87 (31%) such reported cases. Of the 27 cases, six foetal metastases occurred and five of these six infants died.

Our report represents the tenth case of maternal transplacental-transmitted melanoma to the foetus [3–6] and the second case of metastatic lesion on the mastoid reported in the literature till date.

The first case, who presented metastasis to the mastoid and lungs, was described by Valenzano Menada et al. [5].

This patient also had a spontaneous regression of transplacental metastases secondary to maternal melanoma at 6 months of age, after a strong chemotherapy associated with disease progression.

In this paper, we have reported the fourth case of spontaneous regression of metastatic melanoma transferred from the mother to the foetus.

Jeremićet al. [4] reported a pregnancy associated with melanoma and foetal anomalies. Two years later, the child and the mother were disease-free.

Cavell [7] also described a case of transplacental-transmitted metastases from malignant melanoma in a two-and-a-half-year-old child whose mother died 4 days after delivery and was diagnosed with an aggressive metastatic melanoma. After 24 months, the child was disease-free.

The mechanism of spontaneous regression of cancer is still under investigation: operative trauma, infection, and immunologic factors have been reported [8–12].

The pathophysiology of transplacental spread of melanoma is unknown. Factors involved can be the high vascularity of the placenta, placental production of angiogenic and vascular endothelial growth factors, and impaired fetal immune response [13–15].

In a recent study, Pagani et al. suggest that the direct activation of NRP-1 (the coreceptor neuropilin-1) by PlGF (the placenta growth factor) independent of the presence of the VEGFR-1 (the tyrosine kinase receptor) contributes to melanoma aggressiveness [16].

Conventional or adult-type melanoma in children has a genomic profile very similar to adult melanoma. The staging system and management of pediatric melanoma is the same as for adult melanoma. Identification of BRAF and NRAS mutations in pediatric melanoma can facilitate the selection of targeted therapies [17, 18].

Treatment for stage IV metastatic melanoma depends on whether the disease is limited (resectable) or disseminated (unresectable). Resection, if feasible, followed by adjuvant treatment with interferon-alfa or clinical trials including immunoglobulin monoclonal antibodies such as ipilimumab/pembrolizumab and vemurafenib, an inhibitor of BRAF is recommended by National Cancer Institute for limited metastatic disease. Alternatively, limited metastatic disease can by treated with systemic therapy or a short period of observation followed by repeated scans as in our example [19].

## 4. Conclusions

Melanoma involves 8% of the cases of cancer occurring during pregnancy. Foetal metastases are always associated with neoplastic involvement of placenta. Biological characteristics of foetal metastases from maternal cancer need further investigation. For this reason, in case of malignancy during pregnancy, we recommend immunohistological analysis of the whole placenta.

We cannot exclude the possibility of a delayed presentation of the disease due to lack of long-term follow-up data on unaffected children born from mothers with metastatic melanoma Thus, newborns who do not present melanoma at birth should be considered at high risk and undergo close follow-up.
